# A novel ATPase gene, *Ab-atps*, plays an important role in the interaction of rice and white tip nematode, *Aphelenchoides besseyi*

**DOI:** 10.1038/s41598-021-97981-2

**Published:** 2021-09-16

**Authors:** Hong-Le Wang, Chun-Ling Xu, Chun Chen, Shan-Wen Ding, Jun-Yi Li, Si-Hua Yang, Hui Xie

**Affiliations:** Laboratory of Plant Nematology and Research Center of Nematodes of Plant Quarantine, Department of Plant Pathology/Guangdong Province Key Laboratory of Microbial Signals and Disease Control, College of Plant Protection, South China Agricultural University, Guangzhou, People’s Republic of China

**Keywords:** Molecular biology, Plant sciences

## Abstract

Plant kinases containing the LysM domain play important roles in pathogen recognition and self-defense reactions. And it could recognize microbe-associated molecules including chitin and other polypeptides. The white tip nematode *Aphelenchoides besseyi* is a migratory parasitic nematode that infects plant shoots. It is distributed over almost all rice-producing areas and causes up to 50% economic losses. The rice *OsRLK3* gene was a defense-related LysM kinase gene of rice. This study showed that the rice LysM kinase *OsRLK3* could be induced by flg22, jasmonic acid, salicylic acid, and chitin. An interaction gene, *Ab-atps* from *A. besseyi*, was identified by screening the interaction between the rice gene *OsRLK3* and an *A. besseyi* cDNA library using yeast two-hybrid screening. *Ab-atps* is a novel ATP synthase gene with a full length of 1341 bp, coding for 183 amino acids. The mRNA of *Ab-atps* was located in the esophagus and reproductive system of *A. besseyi*. The expression of *Ab-atps* was assessed at different developmental stages of the nematode and found to be the highest in the juvenile, followed by the egg, female, and male. Reproduction was significantly decreased in nematodes treated with *Ab-atps* double-stranded RNA (dsRNA) (*p* < 0.05). Transient expression experiments showed that *Ab*-ATPS-GFP was distributed in the nucleus, cytoplasm, and cell membrane, and *Ab*-ATPS-GFP triggered plant cell death. *OsRLK3* was expressed significantly higher at 0.5 day and 1 day (*p* < 0.05) in rice plants inoculated with nematodes treated with *Ab-atps* dsRNA and *gfp* dsRNA for 0.5–7 days, respectively. Further, *OsRLK3* expression under *Ab-atps* dsRNA treatment was significantly lower than with *gfp* dsRNA treatment at 0.5 day (*p* < 0.05) and significantly higher than with *gfp* dsRNA treatment at 1 day (*p* < 0.05). These results suggest that rice *OsRLK3* could interact with *A. besseyi Ab-atps*, which plays an important role in growth, reproduction, and infection of the nematode. Our findings provide a theoretical basis to further understand the parasitic strategy of *A. besseyi* and its interaction mechanism with host plants, suggesting new ideas and targets for controlling *A. besseyi*.

## Introduction

Rice (*Oryza sativa*) is one of the most important food crops in the world. Several plant nematodes infect rice plants. The economic loss of rice caused by plant nematodes is around 10–25% per year, and white tip nematode, *Aphelenchoides besseyi,* is the most important plant nematode that damage the aboveground parts of rice. *A. besseyi* is a migratory parasitic nematode, which is found in most rice-growing areas of the world. Its life cycle is 7–10 days at 21–25 °C. Upon infection, rice generally present white tips and spikelets, which can cause up to 50% economic loss due to severe damage^1–5^.

Plants are frequently attacked by pathogens and have evolved a multilayer self-defense reactions^6^. Upon pathogen attack, plants respond with production of specific alarm signals salicylic acid (SA), jasmonic acid (JA), bacterial flagellin protein (flg22) and ET, etc., which varies greatly in quantity, composition, and timing, and it is so-called the specificity of the plant’s primary induced defense response. The signaling pathways that are activated upon endogenous accumulation of these signals regulate different defense responses that are effective against partially distinct pathogens^7^.

Plant kinase genes play a key role in pathogen recognition and plant defense responses. When plants are infected by pathogens, kinase receptors can recognize particular signaling molecules of pathogens, which are further transmitted downstream and activate the expression of transcription factors, initiating self-defense mechanisms to regulate the physiological and biochemical processes in the interaction of plants and pathogens^8^. Plant defense response to pathogen infection is regulated by a complex network. When attacked by a pathogen, plants recognize signaling molecules and activate their own defense responses. Using signaling molecules to treat target hosts is helpful for understanding the roles of plant target genes in plant defense responses. Mitogen activated protein kinase (MAPK) and leucine rich repeat receptor-like kinases (LRR-RLK) play an important role in the signal pathways of plant defense reactions, which are induced by the defense signal molecules JA and SA. For example, MAPK kinase *BnOIPK* can be induced to express by JA, but is not sensitive to SA^9^. MAPK kinase *OsSJMK1* and *OsBWMK1* can be induced by JA and SA^10,11^, and LRR-RLK kinase *OsGIRL1, OsSalT*, and *OsPBZ1* can be induced by SA^12^.

Among plant pathogens, there is a particular group of evolutionarily conserved molecules that can induce the defense response of host plants, known as microbe-associated molecular pattern (MAMP). Currently, reported MAMPs include flg22, EF-Tu protein, chitin, and peptidoglycan (PGN)^13^. In plants, there is a particular class of kinase proteins containing a lysine motif (LysM domain). These LysM kinases are mainly recognition receptors, which are located at the cell membrane^14^ and can directly or indirectly recognize MAMPs of pathogens. For example, the plant LysM kinase CERK1, which is found in *Arabidopsis* and rice, can specifically recognize the MAMP chitin and stimulate a self-defense response through chitin-related pathways^15,16^. However, during the long periods of co-evolution between plants and plant pathogens, pathogen gradually evolved an infection mechanism that can inhibit or evade the plant defense response, mediated by LysM kinase protein. For example, AvrPtoB, the effector protein of *Pseudomonas syringae*, can ubiquitinate and degrade LysM kinase CERK1 in *Arabidopsis*, thus inhibiting LysM protein recognition mechanisms in *Arabidopsis*^17^.

More than 1500 kinase genes have been predicted according to the reported rice genome^18^, but there are no reports on the role of rice kinase in the interaction of rice and *A. besseyi*. Wang et al.^5^ reported that the rice kinase gene *OsRLK3* (OS01G0741200) was significantly upregulated and downregulated at the early and late stage of rice infected by *A. besseyi*, respectively. Thus, the authors suggested that *OsRLK3* is involved in the recognition process and could stimulate a self-defense response at the early stage, but might be inhibited by effectors secreted by *A. besseyi* at the late stage during interaction between rice and *A. besseyi*. In this study, interaction genes were screened through yeast two-hybridization between *OsRLK3* and a cDNA *A. besseyi* library. Related functions were studied to explore the role of the rice kinase in the interaction of rice and *A. besseyi*, so as to provide a theoretical basis for uncovering the defense mechanisms of rice against *A. besseyi*. Our findings may provide useful data to study the other interactions of plants and plant migratory parasitic nematodes.

## Results

### Expression levels of *OsRLK3* in rice treated with flg22, JA, SA, and chitin

**Figure 1:**
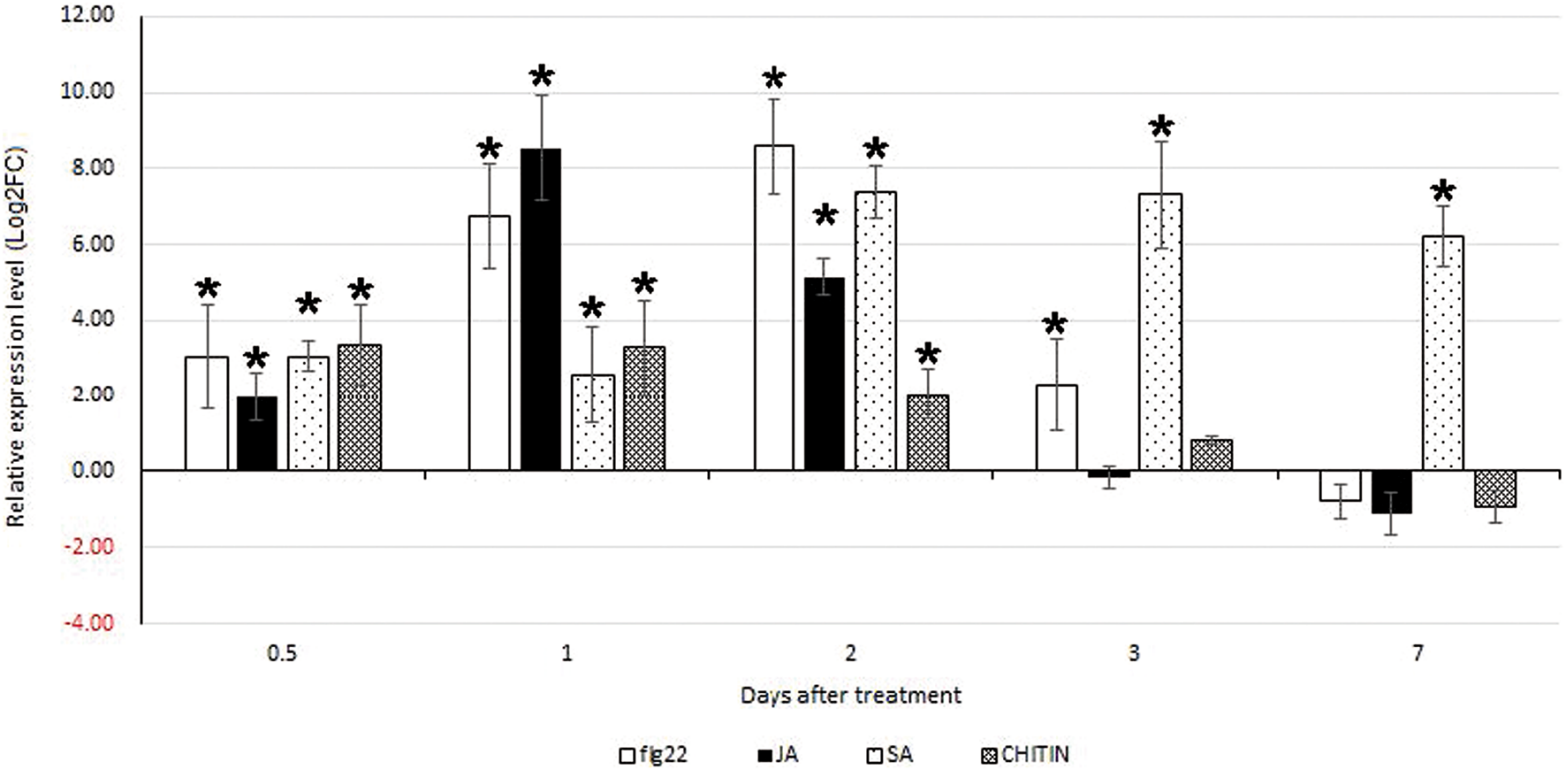
Expression of the receptor-like gene *OsRLK3* upon treatment of rice with flg22, JA, SA, and chitin at different time points. Bars indicated standard errors of mean data (n = 3) from three biological and two technical replicates each containing a pool of three plants. Data are shown as relative expression levels of treated/untreated tissue in comparison with the control tissue. Gene expression levels were normalized using the internal reference gene *OsUBQ5*. The asterisk (*) indicates significant differential expression compared with the control group (p < 0.05). flg22: flagellin 22; JA: jasmonic acid; SA: salicylic acid; CHITIN: chitin.

Expression levels of *OsRLK3* in rice treated with Flagellin protein flg22, JA, SA, and chitin were detected by qPCR. After treatment with flg22, the *OsRLK3* gene was significantly upregulated at 0.5–3 days (*p* < 0.05). The highest level was at 2 days and it was downregulated, but not significantly different to the control, at 7 days (*p* > 0.05). After treatment with JA, the *OsRLK3* gene was significantly upregulated at 0.5–2 days (*p* < 0.05). The highest level was at 1 day and it was downregulated, but not significantly different to the control, at 3 days and 7 days (*p* > 0.05). After treatment with SA, the *OsRLK3* gene was significantly upregulated at 0.5–7 days (*p* < 0.05), and the highest level was at 2–3 days. After treatment with chitin, the *OsRLK3* gene was significantly upregulated at 0.5–2 days (*p* < 0.05). The highest level was at 0.5–1 days and the expression level decreased over time, but was not significantly different to the control at 3 days and 7 days (*p* > 0.05) (Fig. 1). These results showed that the expression of *OsRLK3* could be induced by flg22, JA, SA, and chitin, and was significantly upregulated at the early stage after treatment. According to the results, *OsRLK3* is indicated to be involved in the self-defense reactions of rice, consistent with the report that *OsRLK3* is significantly upregulated at the early stage in rice infected by *A. besseyi*^5^.

### Results of *OsRLK3* cloning and sequence analysis

A full coding cDNA sequence of 2064 bp in length was amplified from rice using the specific primers RP3F and RP3R (Table S1) and confirmed by sequencing. This sequence is 100% similar to the reported *OsRLK3* gene sequence (GenBank accession: OS01G0741200) available in the database of the Rice Genome Annotation Project. This cDNA sequence encodes 687 amino acids, including a signal peptide (between residues 1 and 30) and a transmembrane helix (between residues 261 and 283). The theoretical molecular mass of OsRLK3 was 73.30 kDa, and the molecular mass without the signal peptide was 70.30 kDa. The results of bioinformatic analysis showed that OsRLK3 had the typical characteristics of a LysM kinase family member, including a LysM domain (between residues 175 and 219) and a kinase domain (between residues 376 and 664) (Fig. S1). The predicted location of OsRLK3 was the cell membrane.

### *OsRLK3* of rice interacts with *Ab-atps *of *A. besseyi*

**Figure 2:**
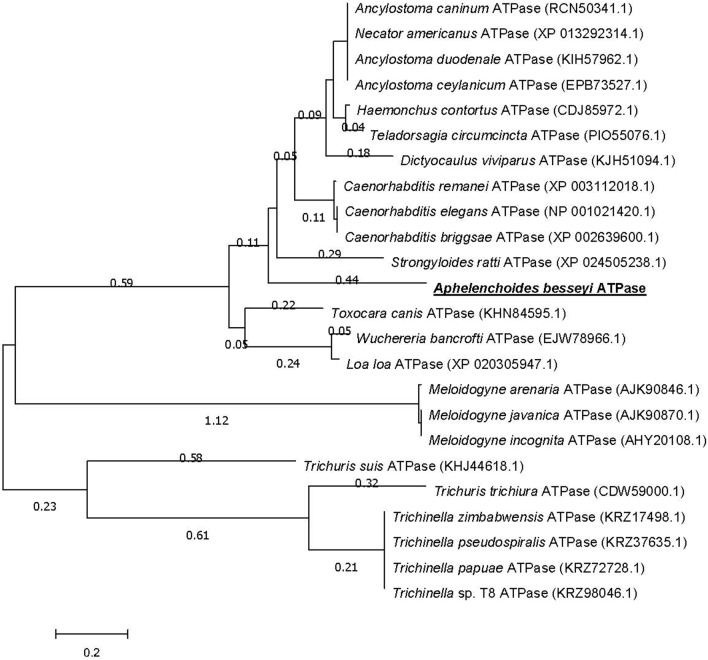
Phylogram constructed on the basis of amino acid sequences depicting the evolutionary relationships among ATP synthase (ATPase) from 24 species of nematodes. *Aphelenchoides besseyi Ab*-ATPS is underlined; Accession numbers of the sequences are shown in brackets; Distances on the *X*-axis correspond to the grade of sequence homology; Distances on the *Y*-axis are arbitrary.

To understand the role of the rice *OsRLK3* gene in the interaction of rice and *A. besseyi*, full coding cDNA of the *OsRLK3* gene was constructed as a bait construct, and used to screen potential interaction genes in a cDNA library of *A. besseyi* using the yeast two-hybrid system. Only one positive clone was identified, and its EST sequence was 301 bp including a poly A in the downstream 3′ untranslated region. Subsequently, a 1341 bp full length cDNA sequence from *A. besseyi* was amplified by RACE (Fig. S2) and confirmed by sequencing. The cDNA sequence included a 552 bp ORF, encoding for 183 amino acids (Fig. S2). The cDNA sequence was identified as *A. besseyi* ATP synthase (ATPase) according to the sequence alignment results of blastx, and named *Ab-atps*, showing highest homology with ATP synthase from *Strogyloides ratti* (GenBank accession: XP_024505238.1, similarity 88%, identity 47.49%, E value:149), followed by ATP synthase from *Necator americanus* (Genbank accession: XP_013292314.1, similarity 86%, identity 45.14%, E value 9e−48), and ATP synthase from *Caenorhabditis elegans* (Genbank accession: NP_001021420.1, similarity 88%, identity 44.69%, E value 4e−47). The phylogenetic tree was constructed based on protein sequences of *Ab*-ATPS and other synthase sequences from 23 species of nematodes in the NCBI database (Fig. 2). The result showed that *Ab*-ATPS has the closest generic relationship with ATP synthase from *Strogyloides ratti,* which was consistent with the results of alignment analysis obtained by blastx. Some sequence had a lower homology with a higher similarity but a lower identity. And it led to some difference in the homology analysis between NCBI Blast and Mega 6.0.

**Figure 3:**
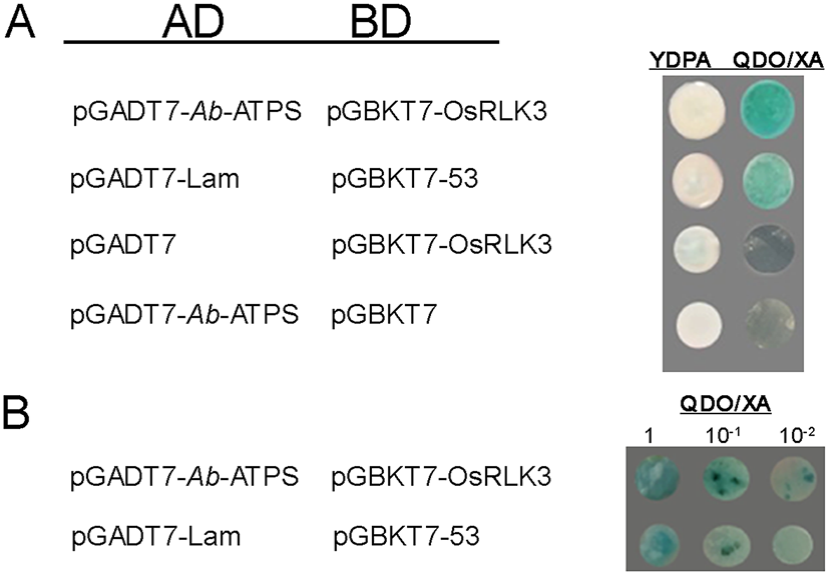
Detection of the interactions between rice *OsRLK3* and *Aphelenchoides besseyi Ab-atps*. (**A**) Interaction of *OsRLK3* and *Ab-atps.* Yeast cells transformed with pGBKT7-OsRLK3 (BD) and pGADT7-*Ab-*ATPS (AD) were grown on YDPA media plates and then printed onto QDO/XA (SD/-Leu/-Trp/-Ade/-His/X-α-gal/AbA) plates, and the colony turned blue; yeast cells transformed with pGADT7-Lam and pGBKT7-53 were used as positive controls; yeast cells were transformed with pGADT7 and pGBKT7-53 were used as negative controls. (**B**) Serial dilutions (1, 10^–1^, 10^–2^) of the yeast cells with pGBKT7-OsRLK3 (BD) and pGADT7-*Ab*-ATPS (AD) were cultured on QDO/XA plates for 1 day to detect their interaction abilities.

The interaction of OsRLK3 and *Ab-*ATPS was tested using the yeast two-hybrid assay. Yeast cells transformed with pGBKT7-OsRLK3 and pGADT7-*Ab*-ATPS exhibited the same blue coloration compared to the positive controls of yeast cells transformed with pGBKT7-53 and pGADT7-Lam. Yeast cells harboring pGBKT7-OsRLK3 co-transformed with the pGADT7 empty vector and those harboring the pGBKT7 empty vector co-transformed with pGADT7-*Ab*-ATPS could not grow on the QDO/XA (SD/-Leu/-Trp/-Ade/-His/X-α-gal/AbA) plate, and did not show interactions. The results showed that *OsRLK3* interacted with *Ab-atps* (Fig. 3).

### Expressions of *Ab-atps *at different developmental stages of *A. besseyi*

**Figure 4:**
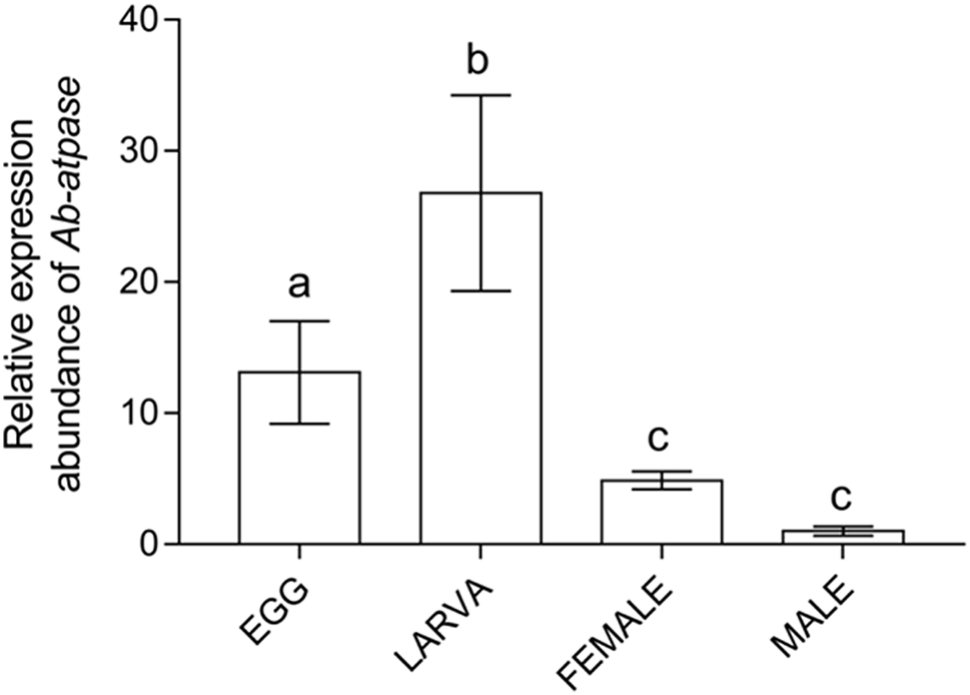
Expression of *Ab-atps* in *Aphelenchoides besseyi* of different developmental stages: egg, juvenile, female, and male. Bars indicate standard errors of mean data (n = 3) and letters indicate significant differences (*p* < 0.05) between treatments.

*Ab-atps* expression levels in eggs, juveniles, females, and males were detected by qPCR. The results showed that *Ab-atps* relative expression level was highest in juveniles, and the expression in juveniles, eggs, and females accounted for 26.79, 13.10, and 4.86 times the expression level in males, respectively (Fig. 4). Significant differences existed among the different developmental stages (*p* < 0.05), but not between females and males (*p* > 0.05).

### In situ hybridization of *Ab-atps*

**Figure 5:**
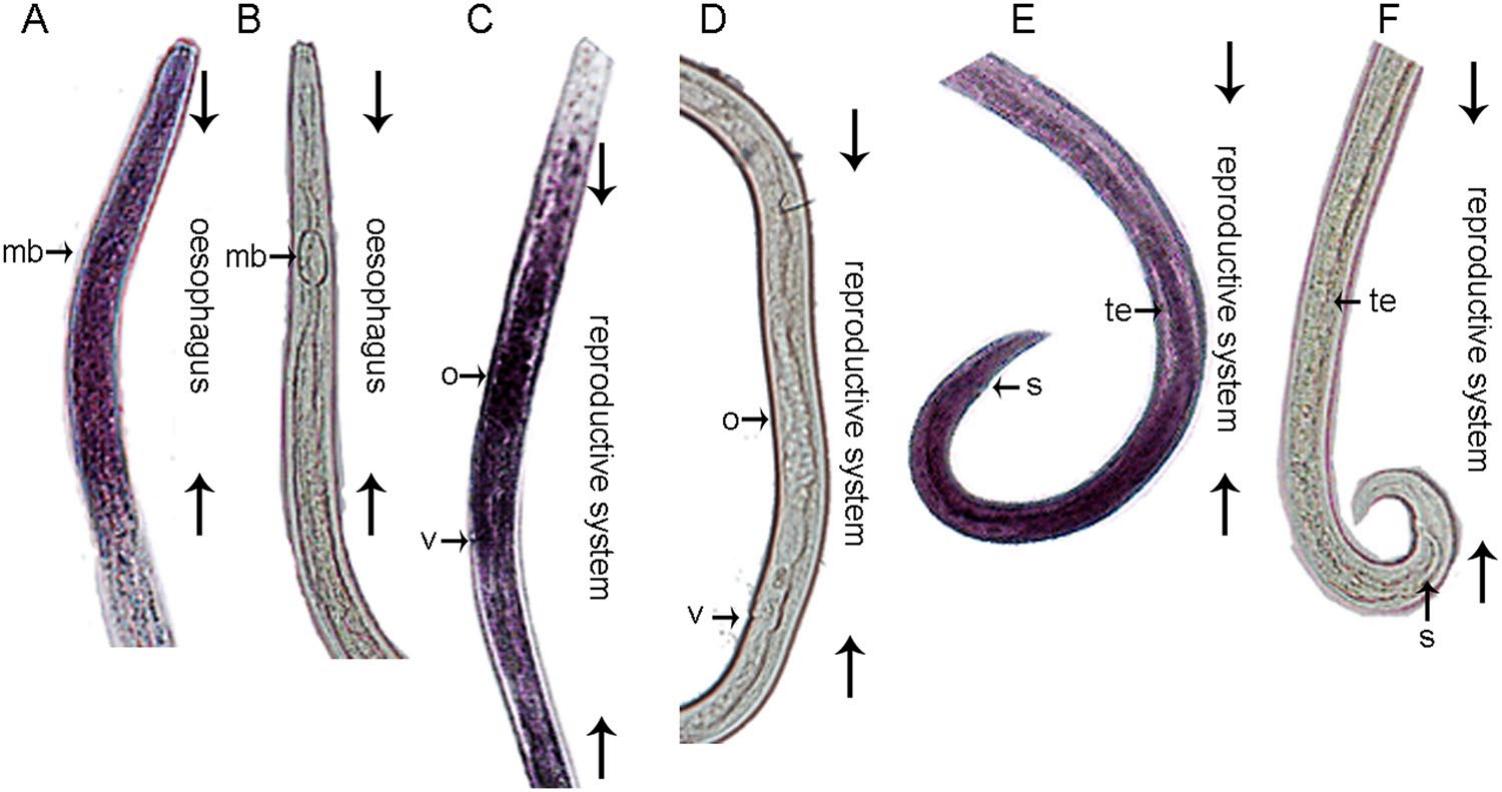
Tissue localization of *Aphelenchoides besseyi* ATPase mRNA *Ab-atps* using in situ hybridization. (**A**) *Ab-atps* mRNA was expressed in the esophagus; (**C**,**E**) *Ab-atps* mRNA was expressed in the reproductive system; (**B**,**D**,**F**) A hybridization signal was observed in the control nematodes using a DIG-labeled sense *Ab-atps* RNA probe; mb: medium bulb, o: ovary, s: spicules, te: testis, v: vulva.

The results of in situ hybridization suggested that *Ab*-*atps* was present in the esophagus and reproductive system (Fig. 5A,C,E). No hybridization signal was detected in nematodes when the control sense *Ab-cb-1* DIG-Labeled RNA probe was used (Fig. 5B,D,F).

### RNAi of *Ab-atps*

**Figure. 6:**
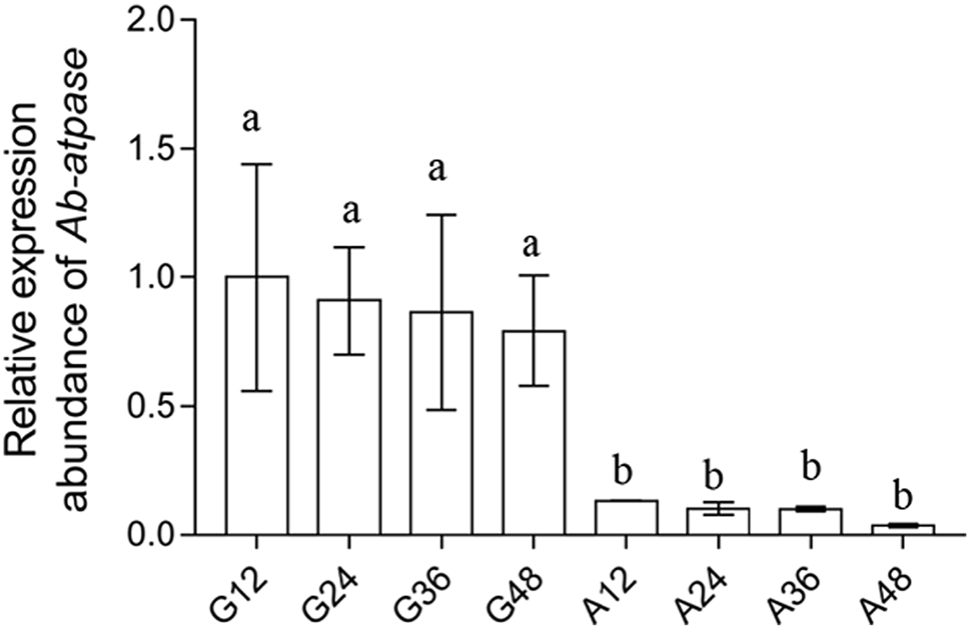
Expressions of the *Ab-atps* mRNA in *Aphelenchoides besseyi* treated with *Ab-atps* double-stranded (ds) RNA. G12, G24, G36, and G48: Expressions of *Ab-atps* mRNA in nematodes that soaked in non-endogenous *gfp* dsRNA solution for 12 h, 24 h, 36 h, and 48 h, respectively; A12, A24, A36, and A48: Expression of *Ab-atps* mRNA in nematodes that soaked in *Ab-atps* dsRNA for 12 h, 24 h, 36 h, and 48 h, respectively. Standard errors of mean data (n = 5) were indicated by bars and significant differences (*p* < 0.05) between treatments were indicated by letters.

After nematodes were treated with *Ab-atps* double-stranded RNA (dsRNA), qPCR was used to detect the RNA interference (RNAi) efficiency of *Ab-atps*. The expressions of *Ab-atps* in these nematodes decreased significantly, when compared to those in treated with the *gfp* dsRNA. And the expression levels were 86.7%, 80.7%, 86.3%, and 75.4%, in nematodes treated with *Ab-atps* dsRNA for 12 h, 24 h, 36 h, and 48 h (*p* < 0.05), respectively. Expression of *Ab-atps* was not significantly different among any of the *Ab-atps* dsRNA treatments (*p* > 0.05), neither among any of the *gfp* dsRNA treatments used as controls in this experiment (*p* > 0.05), respectively (Fig. 6).

**Figure. 7:**
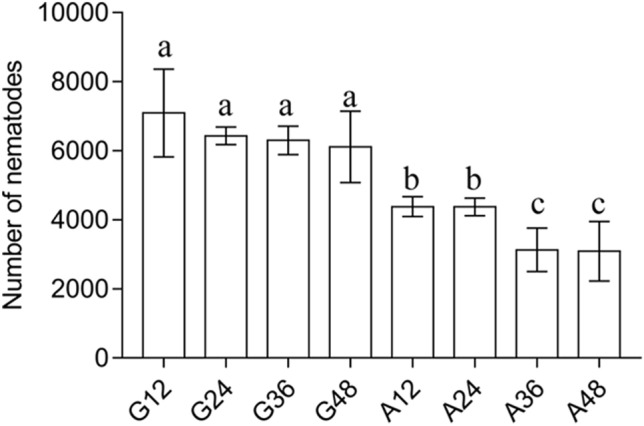
Number of *Aphelenchoides besseyi* on carrot callus after inoculation of 30 females for 35 days. G12, G24, G36, and G48: Number of *A. besseyi* after inoculating 30 females soaked in non-endogenous *gfp* dsRNA solution for 12 h, 24 h, 36 h, and 48 h, respectively; A12, A24, A36, and A48: Number of *A. besseyi* after inoculating 30 females soaked in *Ab-atps* dsRNA for 12 h, 24 h, 36 h, and 48 h, respectively. Standard errors of mean data (n = 5) were indicated by bars and significant differences (*p* < 0.05) between treatments were indicated by letters.

The effect of *Ab-atps* RNAi on the reproduction of *A. besseyi* was examined by culturing the nematodes on carrot disks, which had been soaked in *Ab-atps* dsRNA. After culturing for 35 days, reproductions of nematodes treated with *Ab-atps* dsRNA for 12, 24, 36 and 48 h were significantly lower, than those of nematodes treated with *gfp* dsRNA, respectively (*p* < 0.05) (Fig. 7). With the treatment time of RNAi, reproduction of nematodes decreased, when treated with *Ab-atps* dsRNA. And significant differences (*p* < 0.05) were found between different RNAi treatment time groups except between 12 and 24 h, and 36 h and 48 h (*p* > 0.05). There was no significant difference between *gfp* dsRNA treatment groups (*p* > 0.05). Therefore, *Ab-atps* RNAi inhibited *Ab-atps* expression in *A. besseyi* effectively by soaking the nematodes in *Ab-atps* dsRNA, and *Ab-atps* RNAi depressed the reproduction of *A. besseyi*.

### Expression of *OsRLK3* gene in rice tissue at different times after inoculation with *A. besseyi* treated with dsRNA

**Figure. 8:**
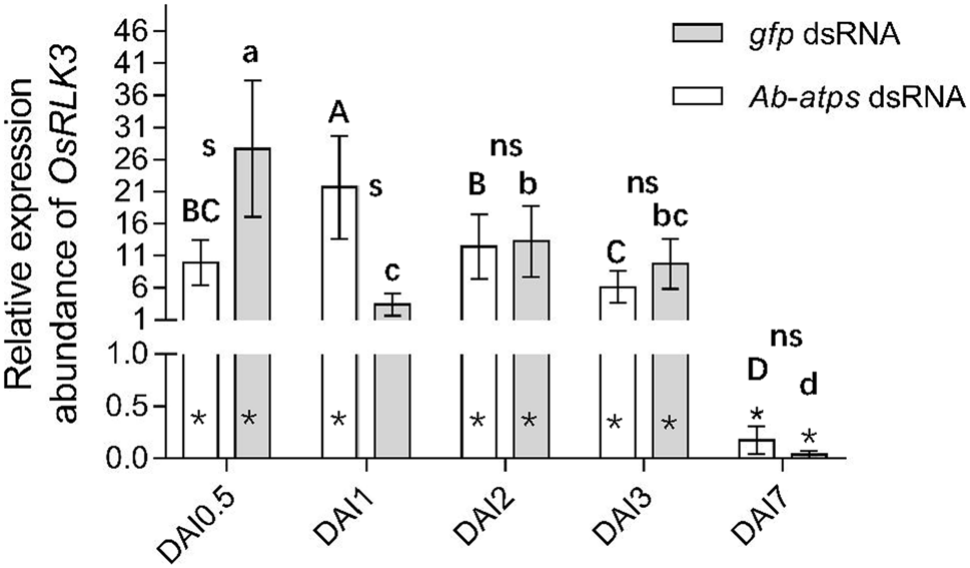
Expression of the *OsRLK3* gene in rice tissue at different times after inoculation with *Aphelenchoides besseyi* treated with dsRNA. Bars indicate standard errors of mean data (n = 3) from three biological and two technical replicates each containing a pool of 3 plants. Data are shown as relative expression levels of treated/untreated tissue in comparison with the control tissue. Gene expression levels were normalized using the internal reference gene *OsUBQ5*. Uppercase letters indicate significant differences (*p* < 0.05) among treatments when rice plants were inoculated with *A. besseyi* treated with *Ab-atps* dsRNA. Lowercase letters indicate significant differences (*p* < 0.05) among treatments when rice plants were inoculated with *A. besseyi* treated with *gfp* dsRNA. The asterisk (*) indicates significant differential expression compared with healthy rice plants (p < 0.05). s: significant difference (*p* < 0.05) between rice plants inoculated with *A. besseyi* treated with *Ab-atps* dsRNA and *gfp* dsRNA at the same time points. ns: not significant (*p* > 0.05) between rice plants inoculated with *A. besseyi* treated with *Ab-atps* dsRNA and *gfp* dsRNA at the same time point. DAI: Days after inoculation; *gfp*: rice inoculated with *A. besseyi* treated with *gfp* dsRNA for 48 h; *Ab-atps*: rice inoculated with *A. besseyi* treated with *Ab-atps* dsRNA for 48 h.

Expression levels of *OsRLK3* in rice shoots were detected at different times (DAI) after rice plants were inoculated with nematodes soaked in *Ab-atps* and *gfp* dsRNA for 48 h. According to the results, the expression of *OsRLK3* gene in rice inoculated with nematodes soaked in *Ab-atps* dsRNA was significantly lower than in those soaked in *gfp* dsRNA at DAI 0.5 (*p* < 0.05), significantly higher than in those soaked in *gfp* dsRNA at DAI 1 (*p* < 0.05), and not significantly different to any soaked in *gfp* dsRNA at DAI 2–7 (*p* > 0.05) (Fig. 8). The highest *OsRLK3* expression was at DAI 1 among those soaked in *Ab-atps* dsRNA (*p* < 0.05), and the highest was at DAI 0.5 among those soaked in *gfp* dsRNA (*p* < 0.05). Therefore, rice inoculated with *A. besseyi* treated with *Ab-atps* RNAi affected the expression levels of the rice *OsRLK3* gene, which indicated its interaction with *Ab-atps*. In addition, expression of *OsRLK3* gene in all treatments was significantly different from that in healthy rice plants (*p* < 0.05), except in rice inoculated with nematodes soaked in *Ab-atps* dsRNA at DAI 1.

### Transient expression of *Ab*-ATPS from *Aphelenchoide besseyi* in tobacco

**Figure. 9:**
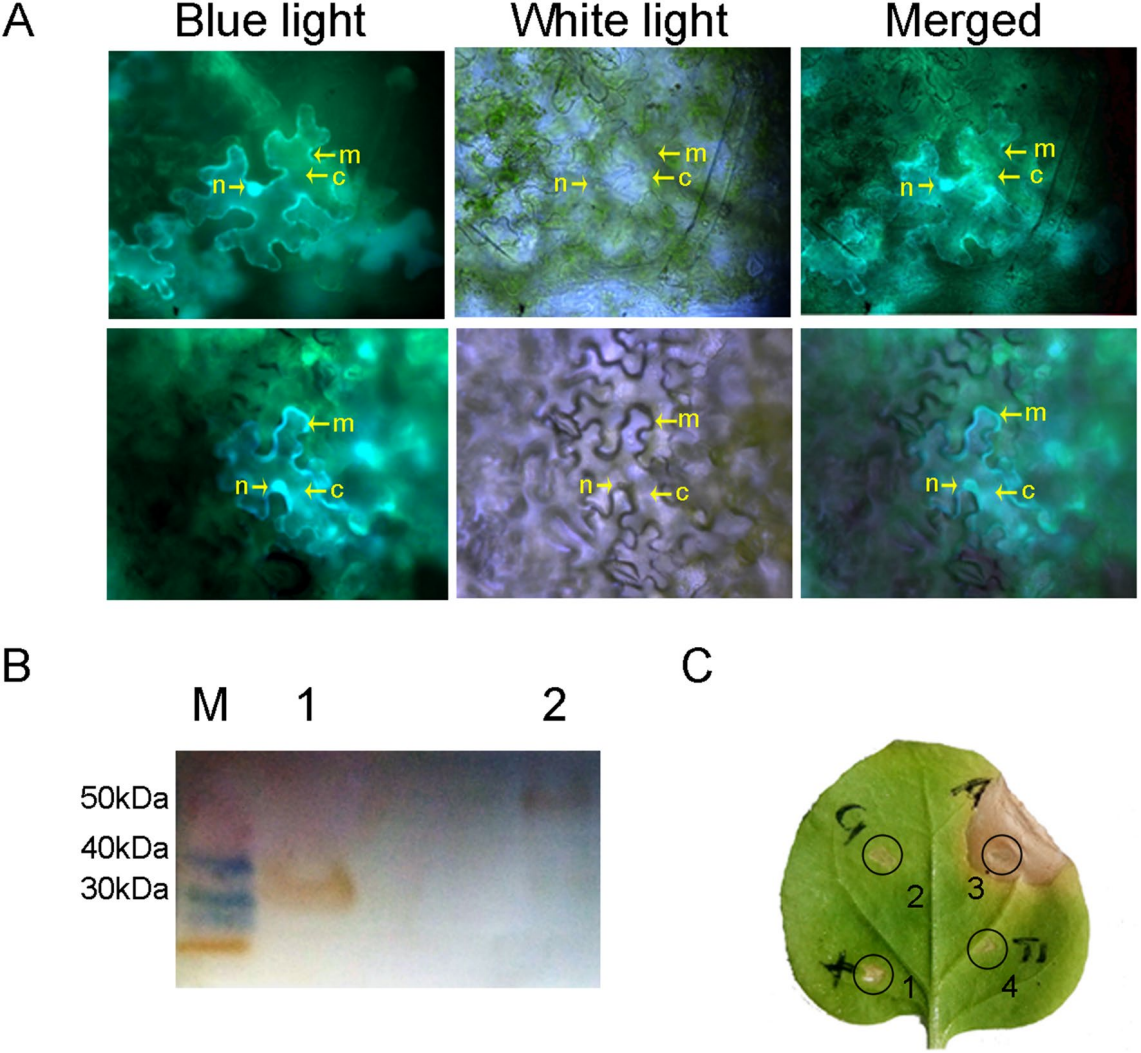
Subcellular localization and transient expression of *Ab*-ATPS from *Aphelenchoides besseyi* in tobacco epidermal cells. (**A**) *Ab*-ATPS-GFP localized at the membrane, plasma, and nucleus in tobacco epidermal cells. (**B**) *Ab*-ATPS-GFP expression was detected by Western-blot. M: Protein Marker (Transgene); 1: Protein mix of GFP extracted from tobacco leaves; 2: Protein mix of *Ab*-ATPS-GFP extracted from tobacco leaves. (**C**) *Ab*-ATPS-GFP triggered cell death. 1: 2-(N-Morpholino) ethanesulfonic acid hydrate (MES) buffer; 2: pCAMBIA1300-GFP; 3: pCAMBIA1300-*Ab*-ATPS-GFP; 4: pCAMBIA1300 empty vector. n: nuclear, c: cytoplasm, m: membrane. GFP: protein was generated with green fluorescent protein.

To confirm the subcellular location of rice OsRLK3 and *A. besseyi Ab*-ATPS in plant cells, full coding cDNA of *Ab-atps* and *gfp* were inserted into the plant transient expression vector pCAMBIA1300 and transiently expressed in tobacco epidermal cells (Fig. 9A). Green fluorescent protein (GFP) and *Ab*-ATPS-GFP localized at the membrane, plasma, and nucleus in tobacco epidermal cells. The recombinant protein extracts were detected by western blot to confirm expression. A distinct band was observed at 40 kDa, which was GFP (approximately 37 kDa), and at 50–70 kDa, which was *Ab*-ATPS-GFP (approximately 57 kDa) (Fig. 9B). At 5 days after infiltration, obvious cell death was observed at the infiltrated site with recombinant *Agrobacterium* of pCAMBIA 1300-*Ab-*ATPS-GFP, compared with no infiltrated sites with pCAMBIA1300-GFP, pCAMBIA1300 empty vector and 2-(N-morpholino) ethanesulfonic acid hydrate (MES) buffer (Fig. 9C). The results confirmed that *Ab-*ATPS from *A. besseyi* triggers plant cell death.

## Discussion

### *OsRLK3* could be induced by chitin, flg22, JA, and SA

The results of this study showed that expression of rice *OsRLK3* could be induced by several signal molecules in the plant defense pathway and *A. besseyi* infection, which means that it plays an important role in the rice defense response to *A. besseyi*. OsRLK3 is a LysM kinase. Most LysM kinases are induced by chitin^10,15^. The LysM kinases Lyp1, Lyk7, and LysMe3, play important roles in chitin perception and defense against *Verticillium dahliae* in cotton. These kinases are induced by chitin, SA, JA, and reactive oxygen species generation, and silencing these genes drastically impairs the defense reactions induced by SA, JA, and reactive oxygen species generation^19^. In the present study, the LysM kinase *OsRLK3* from rice was also induced by chitin, flg22, JA, and SA. These findings hint at a potential involvement of *OsRLK3* in the chitin, flg22, JA signaling pathways, especially the SA signaling pathway because the up-regulation of *OsRLK3* persisted longer when induced by SA than that induced by others. More studies should be carried out in the future to clarify the regulation of *OsRLK3* expression.

### The characteristics and function of *Ab-atps *in *A. besseyi *and its interaction with rice *OsRLK3*

The *Ab-atps* gene interacting with rice *OsRLK3* was identified from *A. besseyi* by yeast two-hybrid screening. We identified this gene as coding for a novel ATP synthase gene, and it was subsequently cloned. The characteristics of this gene were confirmed. Most ATPase genes are expressed in the esophagus, the intestine, the hypodermis, reproduction system, etc^20^. The nematode ATP synthase gene is closely related to the growth, development and reproduction of nematodes, and RNAi of ATP synthase genes led to the death of embryos and diapause of juveniles^20–23^. ATPase expressed in esophagus is essential for survival^19^. The role of ATPase in nematode development is conserved, and it indicated its role in ovulation and reproduction were also conserved^20,24,25^. At present, the ATP synthase genes of pathogenic parasitic nematodes are being studied as targets for drug development^20^. So far, ATP synthase has been reported in more than 10 species of nematodes including animal parasitic nematodes and free-living nematodes^20,26–29^.In plant parasitic nematodes, only the ATP synthase gene of *Meloidogyne incognita* has been reported to be closely related to the pathogenicity of *M. incognita* to host plants^21–23^. In the present study, we found that *A. besseyi Ab-atps* mRNA was located in the esophagus and reproductive system. *Ab-atps* RNAi depressed the reproduction of *A. besseyi,* and rice inoculated with *A. besseyi* treated with *Ab-atps* RNAi affected the expression levels of the rice *OsRLK3* gene. The *Ab-atps* relative expression level was highest in juveniles, followed by eggs, and lowest in adult nematodes. The transient expression of *Ab-atps* in plants triggered cell death. Therefore, the results indicated that *Ab-atps* from *A. besseyi* may be related to the growth, development, reproduction, and infection of the plant nematode *A. besseyi*.

Plants have developed multiple pathways for defense response against pathogens. One example is rapid death of challenged cells upon pathogen attack, leading to the formation of local lesion. This is termed as the hypersensitive response (HR)^30^. If there is an incompatible pathogen–plant interaction, local programmed cell each (PCD) reaction results in mobility arrest, growth retardation and confinement of invading pathogen at the infection site^31^. *N. benthamiana* was a valuable heterologous system for fast-forward analysis of plant pathogens regardless of their host plant^32–35^ with an in planta transient expression assay. The fact that *Ab*-ATPS-GFP localized at the membrane, plasma, and nucleus in tobacco epidermal cells indicated that *Ab-atps* is recognized by the host plant, possibly inducing plant self-defense responses. The ATPase gene is related to plant cell death. The ATPase gene *PDE1* of *Magnaporthe grisea* was closely related to appressorium formation under infection^36^. Another ATPase gene, *MgAPT2* from *M. grisea*, induces plant resistance and triggers cell death^37^. Plants also activate self-defense responses by self-secreting ATPase. Lee and Sano^38^ reported that tobacco ATPase NtAAA1 participates in the self-defense response, and after its silencing, the anaphylactic cell necrosis defense response of tobacco was inhibited by inoculation with the pathogen *Pseudomonas syringae*. The AAA ATPase *AtOM66*, localized in the mitochondrial membrane of *Arabidopsis thaliana*, plays an important role in triggering cell death to resist pathogen infection^39^. In our study, we also found a similar result in that the ATP synthase gene *Ab-atps* from *A. besseyi* could induce plant cell death in tobacco. This indicates that *Ab-atps* can be recognized by the host plant and is involved in the plant defense response in the interaction of rice and *A. besseyi*. Based on these results, *Ab-atps* might be important for developing new methods to control *A. besseyi*.

### Rice *OsRLK3* interacts with *A. besseyi Ab-atps*

The expression of *OsRLK3* gene was affected by *A. besseyi Ab-atps.* At DAI 0.5, *OsRLK3* expression was highest in rice inoculated with nematodes soaked in *gfp* dsRNA (*p* < 0.05), and was significantly higher than that of rice inoculated with nematodes soaked in *Ab-atps* dsRNA (*p* < 0.05). Meanwhile, *OsRLK3* expression was highest in rice inoculated with nematodes soaked in *Ab-atps* dsRNA (*p* < 0.05) at DAI 1. The expression of *OsRLK3* was induced by flg22, SA, JA, and chitin. The highest upregulation of *OsRLK3* was observed at 0.5–1 days in rice treated with JA and chitin, and at 2 day and 2–3 days in rice treated with flg22 and SA, respectively. These results indicate that *Ab-atps* might be related to the rice defense induced by chitin and JA, which are initiated by *A. besseyi* attack. Rice *OsRLK3* was able to recognize *A. besseyi Ab-atps* and activated a self-defense response. However, the regulation of *OsRLK3* is complex, as it was induced by several signaling molecules and further study is needed. Many studies have shown that LysM kinases recognize MAMP molecules of pathogen, and stimulate self-defense responses through chitin-related pathways, including CERK1 of rice and *Arabidopsis*; LYP4 and LYP6 of rice; and AtLYK1, AtLYK4, AtYLK5, and AtLYP1-3 of *Arabidophsis*. LysM kinases are also the target of pathogen effectors^14–16,40^. Wang et al.^5^ reported that *OsRLK3* was significantly upregulated at the early stage and downregulated at the late stage of rice infected by *A. besseyi* through transcriptome sequencing, and our study confirmed these results. Therefore, we speculate that through the interactions of rice and *A. besseyi*, *OsRLK3* recognizes *Ab-atps* and stimulates self-defense responses at the early stage of infection, although *A. besseyi* may secrete effectors to suppress *OsRLK3* at the late stage, given that *OsRLK3* was inhibited at the late stage after infection in our study. However, the mechanism by which *A. besseyi* inhibited or evaded the defense response mediated by *OsRLK3* requires further study. In addition, it has been reported that nematode ATPase showed much similarity and share conserved domains^20,29^. The interaction between *OsRLK3* and *Ab-atps* provide some insight into understanding the interaction mechanism between rice and nematode *A. besseyi.*

## Conclusions

Our results suggest that rice *OsRLK3* could interact with *A. besseyi Ab-atps*, which plays an important role in growth, reproduction, and infection of the nematode. Our findings provide a theoretical basis to further understand the parasitic strategy of *A. besseyi* and its interaction mechanism with host plants, suggesting new ideas and targets for controlling *A. besseyi*. Our finding also suggested new ideas and targets for developing new methods to control *A. besseyi*.

## Methods

### Plants materials, nematode, vectors, and chemically competent cell strains

Rice, *Oryza sativa* L. cv. “Nipponbare”, was obtained from Prof. Guohui Zhou of the Laboratory of Plant Virus, South China Agricultural University, and cultivated as described by Wang et al.^5^. Tobacco, *Nicotiana benthamiana,* was preserved in the laboratory, and cultivated as described by Li et al.^41^ and Wang et al.^4^. The *A. besseyi* used in this study was collected from rice, *O. sativa,* in Luhe Town, Nanjing, Jiangsu Province, China. Nematodes were isolated, identified, preserved, and cultured by the Laboratory of Plant Nematology, South China Agricultural University. The preservation and cultivation methods for nematodes were as described by Cheng et al.^42^. As for the vectors, pMD-18T was purchased from Takara (Shiga, Japan); pGBKT7 and pGADT7 were purchased from Clontech (CA, USA); and pCAMBIA1300 was preserved in the laboratory. The *Escherichia coli* chemically competent cell strain DH5α was purchased from Transgene Biotech (Beijing, China). The yeast (*Saccharomyces*) chemically competent cell strains Y2H and Y187, and the *Agrobacterium tumefaciens* chemically competent cell strain GV1301 purchased from Shanghai Weidi Biotechnology (Shanghai, China). All test materials were used under approved protocols and guidelines at South China Agricultural University.

### Expression levels of *OsRLK3* in rice treated with flg22, JA, SA, and chitin

At the two-leaf stage, healthy rice plants were treated with flg22, JA, SA, and chitin respectively. Flagellin protein flg22 (amino acid sequence, QRLSTGSRINSAKDDAAGLQIA) and chitin were purchased from Sangon Biotech Co., Ltd (Shanghai, China). SA with purity ≥ 99.5% was purchased from Tianjin Baishi Chemical Co., Ltd (Tianjin, China). JA with purity ≥ 85% was purchased from Shanghai Makclin Biochemical Co., Ltd (Shanghai, China). At the two-leaf stage of rice, 1 ml of 2-μM flg22 protein water solution was infiltrated into the surface of rice plant^43^; for SA, JA, and chitin treatments, rice plants were sprayed with 100 μM SA, JA, and chitin water suspension separately and evenly^10,44^. The water used for solution preparation had been sterilized before use. The treated rice plants were grown in the growth chamber at 30 °C. They were watered once a week, and were under a 16 h: 8 h light: dark regime, 150 μmol/m^2^/s^1^ light density, and 70–75% relative humidity.

Rice shoots treated by flg22, JA, SA, and chitin for 0.5, 1, 2, 3, and 7 days were used for RNA extraction. The expression pattern of *OsRLK3* was detected. RNA extraction was conducted using the RNAprep Pure Plant Kit (Tiangen, Beijing, China). The extracted RNA was diluted to 100 ng/μl using RNase-free water as a template for cDNA reverse transcription, after examination by electrophoresis for integrity and Nanodrop spectrophotometer for purity. Reverse transcription was performed following the instructions of the HiScript Q RT SuperMix for qPCR (+ gDNA wiper) kit (Vazyme, Nanjing, China). The relative expression levels of target defense-related genes in rice at different times were detected by qPCR using the reverse transcribed cDNA as a template^5^. The primers of target genes *OsRLK3* and *OsUBQ5* that were used are shown in Table S1. Each treatment included three biological replicates composed of three rice plants. All PCRs were performed in two technical replicates. qPCRs were performed in a CFX96 (Bio-Rad, CA, USA), and data were analyzed using the Bio-Rad CFX 96 Manager (Version 1.5 534.0511) and REST 384 software^45^.

### Cloning of *OsRLK3* and sequence analysis

RNA from rice plants was used as a template and reverse-transcribed into cDNA using the PrimeScript II 1st Strand cDNA Synthesis Kit (Takara). Primers RP3F and RP3R (Table S1) were designed for the full coding cDNA amplication of the *OsRLK3* gene (GenBank accession: OS01G0741200) according to its sequence in the database of the Rice Genome Annotation Project. Sequence analysis was performed using DNAman 6.0 (Lynnon Biosoft, CA, USA). Protein bioinformatic analysis was performed using Protein Machine software (http://www.expasy.ch/tools/), including predictions of protein transmembrane region, amino acid sequence, isoelectric point analysis, molecular weight and hydrophobicity analysis. Predictions for signal peptide and cleavage site analysis were performed at http://www.cbs.dtu.dk/services/SignalP/. Cell location analysis was performed at http://psort.ims.u-tokyo.ac.jp/form2.html.

### Yeast two-hybrid screening

#### Construction of pGBKT7 recombinant

The yeast two hybrid system (Clonech) was used for screening interaction genes between rice kinase *OsRLK3* and a cDNA library of *A. besseyi*. According to the protocol described for the ClonExpress II One Step Cloning Kit (Vazyme), full coding cDNA of the *OsRLK3* gene was connected to the DNA binding domain (BD) of pGBKT7. The full coding cDNA of *OsRLK3* gene obtained in previous *OsRLK3* gene cloning was used as the template, primers BDRP3F and BDRP3R (Table S1) were used for amplification, and the restriction enzymes used were NcoI and BamHI. According to the protocol of the Matchmaker two-hybrid system (Clontech), the full coding region of *OsRLK3* was cloned into the GAL4 binding domain vector pGBKT7 as a bait construct after sequence confirmation, and was transformed into the Y2H yeast strain.

#### Construction of the *A. besseyi* cDNA library

Total RNA of approximately 20,000 mixed-stages nematodes were extracted using the Invitrogen TRIzol Reagent kit (Invitrogen, Carlsbad, CA, USA). The cDNA library of *A. besseyi* was constructed in the GAL4 activation domain vector pGADT7 according to the manufacturer’s protocol, and transformed into the Y187 yeast strain.

#### Yeast two-hybrid screening

The Y2H and Y187 yeast strains were co-transformed. The transformants were screened according to the manufacturer’s protocol. Interaction screening was carried out on QDO (SD/-Leu/-Trp/-Ade/-His) and QDO/XA plates. The blue colored clone, which was cultured on the QDO/XA plate for 3–5 days at 30 °C, was considered to have an interaction with rice kinase OsRLK3. Plasmids of positive clones were extracted using the HiPure Yeast Plasmid Mini Kit (Magen, Guangzhou, China), transferred into *E. coli* DH5α competent cells, and selected for sequencing (BGI Company, Shenzhen, China) using primers T7 and 3′AD (Table S1).

#### Cloning of the full-length *Ab-atps *gene from *A. besseyi*

Total RNA of nematodes was reverse transcribed into cDNA using the BD SMARTTM PCR cDNA Synthesis Kit (Takara). 5′ RACE primers (IA-D2R, D2RACER1) (Table S1) were designed for cDNA amplification according to the sequencing results of positive clones selected from the *A. besseyi* cDNA library. The amplified products were purified and ligated with pMD 18-T vector (Takara) to obtain recombinant plasmid. Recombinant plasmid was transformed into *E. coli* DH5α competent cells, and then positive clones were selected for sequencing (BGI Company) as described method by Cheng et al.^42^. According to the sequencing results, primers D2F and D2R (Table S1) were designed for the full-length amplification of the *Ab-atps* gene from *A. besseyi*.

#### Sequence analysis, alignment and phylogenetic analysis of *Ab*-ATPS

Sequence homology alignments were performed using NCBI blastn and blastx (http://blast.ncbi.nlm.nih.gov/Blast.cgi). Predictions of signal motifs and cleavage sites were performed at http://www.cbs.dtu.dk/services/SignalP/. Predictions of transmembrane regions were performed at http://www.cbs.dtu.dk/services/TMHMM/#opennewwindow. The phylogenetic tree was constructed using the neighbor-joining method^46^ using the program MEGA 6.0 (Molecular Evolutionary Genetics Analysis, USA) based on *Ab*-ATPS and other 23 ATP synthase sequences of representative nematodes in the NCBI database.

### Confirmation of the interaction between OsRLK3 and *Ab*-ATPs

The full coding region of *Ab-atps* was cloned into the GAL4 activation domain vector pGADT7, according to the protocol of the ClonExpress II One Step Cloning Kit (Vazyme). The primers used were ADD2F and ADD2R (Table S1), and the restriction enzymes used were EcoRI and BamHI. The recombinant plasmid was extracted as a prey construct after sequencing (BGI Company). According to the instructions of Matchmaker two-hybrid system (Clotech), the Y2H yeast strain was co-transformed with the pGADT7-*Ab*-ATPS and pGBKT7-OsRLK3 vectors. Yeast cells were co-transformed with vectors pGBKT7 and pGADT7-*Ab*-ATPS, and vectors pGBKT7-OsRLK3 and pGADT7 were used as negative controls. Yeast cells co-transformed with pGBKT-Lam and pGADT7-53 served as positive controls. Interaction screening was carried out on QDO plates and QDO/XA plates. The blue colored clone, which was cultured on QDO/XA plates for 3–5 days at 30 °C, was considered the bait construct that interacted with the prey constructs.

### Expression of *Ab-atps *at different development stages of *A. besseyi*

RNA of different developmental stages was extracted from 500 each of females, males, juveniles, and eggs of the nematode using MicroElute total RNA kit (OMEGA, GA, USA), respectively. The extracted RNA was reverse transcribed into cDNA using the RQ1 Rnase-Free Dnase (Promega, WI, USA) reverse transcription kit as described above. The expression levels of *Ab-atps* in four development stages were detected on a CFX-96 (Bio-Rad) qPCR machine with cDNA as a template, using the SYBR Green Real-time PCR Master Mix-plus kit (TOYOBO, Osaka, Japan). Specific primers QD2F and QD2R (Table S1) were designed to detect *Ab-cb-1* expression. The 140 bp of 18S rRNA (AY508035) was amplified as a reference gene using the primers Ab18sF and Ab18sR (Table S1). qPCR data was analyzed using CFX manger software provided by Bio-Rad. All experiments were performed in three replicates.

### In situ hybridization of *Ab-atps*

In situ hybridization was performed as described as De et al.^47^. Approximately 10,000 nematodes of mixed-stages of *A. besseyi* were collected and concentrated into 30–50 μL. The nematodes were fixed in 3% paraformaldehyde for 18 h at 5 °C and later at 22 °C for 4 h. DIG-labelled sense and antisense RNA probes (Roche, Mannheim, Germany) were synthesized using sense primers (IS-D2F, IS-D2R) and anti-sense primers (IA-D2F, IAD2R) (Table S1) and the full-length cDNA of *Ab-atps* as a template. DIG-labeled RNA probes were added to the hybridization solution containing nematode and then rotated for 12 h at 47 °C. Results were examined and photographed by an optical microscopy (Nikon Eclipse 90i, Nikon, Japan).

### dsRNA synthesis and RNAi efficiency of *Ab-atps*

RNA interference (RNAi) of the *Ab-atps* gene was carried out by soaking the nematodes with dsRNA of *Ab-atps* synthesized by in vitro transcription. Two primer pairs of IS-D2F/ISD2R and IA-D2F/IA-D2R (Table S1) were designed to amplify the sense and antisense single-stranded RNA (ssRNA) products. *Ab-atps* dsRNA was synthesized according to the instructions of the Script MaxTM Thermo T7 Transcription kit (TOYOBO). The obtained dsRNA synthesis product was purified using the previously described method^42^, then examined for integrity by electrophoresis, detected for concentration and quality using a Nano-drop spectrophotometer, and stored at − 80 °C until further use. The non-endogenous control dsRNA (125 bp) (green fluorescent protein gene, *gfp*) was generated with using the specific primers G-T7S, G-A, and G-S (Table S1)^48^. Five hundred mixed-stages nematodes were separated from carrot callus and collected in a DEPC-treated centrifuge tube. 50 μl of *Ab-atps* dsRNA (2 μg/μL) was added into to the tube for soaking the nematodes at 25 °C. The nematodes were soaked for 12, 24, 36, and 48 h. Non-endogenous *gfp* dsRNA solution (50 mL; 2 μg/μL) was used as a control. There were total of eight treatments, all performed in triplicate. RNA of nematodes treated with dsRNA solution soaking was extracted after washing the nematodes three times with DEPC water. RNAi efficiency was examined through determination of *Ab-atps* expression levels by qPCR.

### Effect of *Ab-atps* RNAi on nematode reproduction

Female nematodes were treated with *Ab-atps* dsRNA for 12, 24, 36, and 48 h, and also treated with *gfp* dsRNA as a control. Thirty female nematodes were selected from each treatment and inoculated on carrot callus, and each treatment was repeated five times. Carrot callus dishes inoculated with nematodes were incubated at 25 °C in the dark for 35 days, then nematodes on carrot callus were separated and counted.

### Expression of *OsRLK3* gene in rice tissue at different times after inoculation with *A. besseyi* treated by dsRNA

Rice plants were inoculated with nematodes of mixed stages treated with *Ab-atps* dsRNA and *gfp* dsRNA for 48 h. Control treatments were healthy plants that were inoculated with 50 μL sterilized water. The inoculation method was as previously described by Wang et al.^5^. Rice shoots were collected at 0.5, 1, 2, 3, and 7 days after inoculation (DAI). RNA extraction, cDNA reverse transcription and *OsRLK3* expression determination were carried out as described in the previous section. Each DAI treatment included three biological replicates and each replicate was composed of three rice plants. All PCRs were performed in two technical replicates.

### Transient expression of rice OsRLK3 and *A. besseyi Ab*-ATPS in tobacco

According to the protocol described in the ClonExpress II One Step Cloning Kit (Vazyme), full coding cDNA of *gfp* was amplified using primers GF/GR (Table S1) and inserted into vector pCAMBIA1300 after SacI/BamHI digest. Full coding cDNA of *Ab-atps* was amplified using primers PD2F/PD2R (Table S1), and inserted into pCAMBIA1300-GFP after SalI/PstI digest. Recombinant plasmids were transformed into *A. tumefaciens* GV3101 by N_2_ transformation^49^ after sequence confirmation (BGI company). Positive clones were screened by LB plates containing both 50 μg/mL kanamycin and rifampicin, and cultured for 2 days at 28 °C. Growth of recombinant *Agrobacterium* and vacuum infiltration of tobacco leaves was performed as previously described^50^. Cell death was observed in tobacco leaves infiltrated with recombinant *Agrobacterium* of pCAMBIA 1300-*Ab-*ATPS-GFP, pCAMBIA1300-GFP, pCAMBIA1300 empty vector and MES buffer. Total protein was extracted using a plant protein extraction kit (KeyGen Biotech, Nanjing, China), separated by 10% SDS–polyacrylamide gel electrophoresis and detected by western blot with green GFP antibody (Transgene).

### Statistical analysis

Data in this study were subjected to analysis of variance (ANOVA) and multiple comparisons of means were conducted using Duncan’s Multiple Range Test at *p* = 0.05 using SAS (Release 8.01), including expression levels of qPCR and nematodes separation counting.

### Ethics approval and consent to participate

Animals were treated in strict accordance with the Animal Ethics Procedures and Guidelines of the People’s Republic of China. All animal procedures were approved by the Animal Ethics Committee of the South China Agricultural University. Plant materials including the collection were used under approved protocols and guidelines at South China Agricultural University.

## Supplementary Information

Supplementary Information.

## Data Availability

All data generated or analyzed during this study are included in this published article and its supplementary information files.
