# Supplementary material for: A novel ATPase gene, Ab-atps, plays an important role in the interaction of rice and white tip nematode, Aphelenchoides besseyi
Source: Sci Rep. 2021 Sep 16;11:18521. doi: 10.1038/s41598-021-97981-2 (PMC8446066; doi:10.1038/s41598-021-97981-2)
Supplement: Supplementary file 1 — Supplementary Information. [file 41598_2021_97981_MOESM1_ESM.pdf]

A novel ATPase gene, *Ab-atps*, plays an important role in the interaction of rice and white tip nematode, *Aphelenchoides besseyi*

Hong-Le Wang, Chun-Ling Xu, Chun Chen, Shan-Wen Ding, Jun-Yi Li, Si-Hua Yang, Hui Xie*

Laboratory of Plant Nematology and Research Center of Nematodes of Plant Quarantine, Department of Plant Pathology / Guangdong Province Key Laboratory of Microbial Signals and Disease Control, College of Plant Protection, South China Agricultural University, Guangzhou, People's Republic of China.

* Corresponding author: Prof. Hui Xie

Email: xiehui@scau.edu.cn

Tel: 00862038297286

ORCID ID: 0000-0002-5035-4427

Supplementray data

Table S1 Primers used in this research

Figure S1 *OsRLK3* cDNA sequence and its deduced amino acid sequence

Figure S2 *Ab-atps* cDNA sequence and its deduced amino acid sequence

Figure S3 Figure of western blots used in Figure 9

Table S1 Primers used in this research

**Primer**	**Sequence (5’-3’)**
RP3F	TGACAACCCCTACCCTAGAGC
RP3R	TGCCAATAGTAAAGTGCAAG
BDRP3F	AGGACCTGCATATGGCCATGGGATGACGCCGCCCCCGGC
BDRP3R	CCGCTGCAGGTCGACGGATCCTCACCTCCCAGCCACAAG AC
T7	TAATACGACTCACTATAGGG
3’AD	AGATGGTGCACGATGCACAG
D2F	TTTGAGCGAAATGTCCGCAAG
D2R	AGTCTACGAGTTCCTACTGACGA
D2RACER1	ATCGCCAATCTGAACGATCAAGCCGCCCAT
TGD2F	TAATACGACTCACTATAGGGAAACAGTATTCATGTCCCCT ATACTAGGT
TGD2R	TTAAACGGCCTCTTTGATGG
ADD2F	GCCATGGAGGCCAGTGAATTCTCCGCAAGTCGTGCCGCC
ADD2R	CAGCTCGAGCTCGATGGATCCAACGGCCTCTTTGATGGTC TC
UPM	CTAATACGACTCACTCACTATAGGGC
NUP	AAGCAGTGGTATCAACGCAGAGT
QD2F	ACTTCGAGGACATTCTCCGTT
QD2R	CATGATCTCCGGTCGAACCTG
Ab18sF	CTCGTGGTGGCTGGTATGCTG′
Ab18sR	GTTTCCCGTGTTGAGTCAAATTAAG
IA-D2F	TAATACGACTCACTATAGGGAAATACGCGACCAGTTTGTAT CAGG
IA-D2R	CCGACTGTTTCAAGTTGCGAT
IS-D2F	TAATACGACTCACTATAGGG CCGACTGTTTCAAGTTGCGAT
IS-D2R	AAATACGCGACCAGTTTGTATCAGG
PD2F	ATCGAGCTCATGGTGAGCAAGGGC
PD2R	CGCGGATCCCTTGTACAGCTCGTC
GF	CGCGTCGACATGTCCGCAAGTCGT
GR	TGGCTGCAGTTAAACGGCCTCTTTGAT

```
1     ATGCCGCCTCCCCGCCGCCGCCCGCCCGCGCCCGGGCTTCTCATCCTCCTCATCTTCCTGGCGCTCGCCTCCCCC
1     M P P P R R R P P A P G L L I L L I F L A L A S P
76    GGCGAAGTCGCCGGTGATGACGCCGCCCCCGGCAATGTCACCGCCTCGGCGCCGCTCCCCTGCTCCGAGCTCTCG
26    G E V A G D D A A P G N V T A S A P L P C S E L S
151   CGCGTCTGCACGGCGTTCGTCGCGTTCCCGGCGGCCGGCGAGGCCGCGAACGCCTCCGTGCTGGAGTCCATGTTC
51    R V C T A F V A F P A A G E A A N A S V L E S M F
226   GACGCCGCGCCGGGGGACATCACGGCCGACGCGGCCGCCAGCCCCGGGTACGCGTTCGTCCGCAAGAACTGCTCC
76    D A A P G D I T A D A A A S P G Y A F V R K N C S
301   TGCCTCGCCTCCCGCACATACCTCGCCAATACGACCTACACTGTCCCCTCCGCCGCCGCCGGGGCGACGGCGAAC
101   C L A S R T Y L A N T T Y T V P S A A A G A T A N
376   GCCACGGCGGCCGACGTGGCTGCCGCGGCGTACGCGGGGCTAGCCGTGCCGCCGCCGGGCGGACCGGCTCTGCGG
126   A T A A D V A A A A Y A G L A V P P P G G P A L R
451   CCGCCGCGCCCCGGCGCCGTGGTGGCTCTCCACCTGCTCTGTGGCTGCTCATCCGGCCCCTGGAACTATCTCCTC
151   P P R P G A V V A L H L L C G C S S G P W N Y L L
526   AGCTACGTCGGCGTCGACGGAGACACGGTGCAATCGCTATCAAGTCGGTTCGGGGCCAGCATGGACGCCATCGAG
176   S Y V G V D G D T V Q S L S S R F G A S M D A I E
601   GCCGCCAATGGCATGGCCGGCCCTGATCCTATAACCACGGGAAAGGTGTACTACATCCCACTCAACTCAGTTCCT
201   A A N G M A G P D P I T T G K V Y Y I P L N S V P
676   GGACAACCATATCCCGTGATATCTCCTTCCTCTCCTACTCCTGCGCCAGCACCCGCACAAAATACATTCTCAGAT
226   G Q P Y P V I S P S S P T P A P A P A Q N T F S D
751   GTATCAGAACATCATTCCACAAAATTCCCTTATGGATGGGTTATTGGCAGCATGGGAGTTGCACTTGCTTTGATT
251   V S E H H S T K F P Y G W V I G S M G V A L A L I
826   GCTATTGCTCTCCTTGCACTTGTTCTGTGCAAGCTCTCCCAATACAATCCCCAAGCTCCAAATAACCAGGGAAAG
276   A I A L L A L V L C K L S Q Y N P Q A P N N Q G K
901   AGCCCAGACCAGTCTATATCACACAAGTTTCAACTTCTTAAGAGTGGTAGTTTTTGTTATGGTTCTGGAAGATAC
301   S P D Q S I S H K F Q L L K S G S F C Y G S G R Y
976   TTATGTTGCCAATTTGGAAATGTGAAACAATCAAGAACGGATGGCAGTGATCATCATATGAATACCCCAAAAGGT
326   L C C Q F G N V K Q S R T D G S D H H M N T P K G
1051  GTTGTAGTGGACGTGTTTGATAGGGAGAAGCCCATTGTGTTTACCTATCAAGAAATACTTGCATCAACCGATTCA
351   V V V D V F D R E K P I V F T Y Q E I L A S T D S
1126  TTTTCTGATGCAAATCTTCTGGGTCATGGTACATATGGCTCTGTTTATTATGGTGTTCTTCGAGACCAGGAGGTC
376   F S D A N L L G H G T Y G S V Y Y G V L R D Q E V
1201  GCTATAAAGAGAATGACAGCAACTAAAACTAAAGAGTTTATAGTGGAGATGAAAGTTCTTTGTAAGGTCCATCAT
401   A I K R M T A T K T K E F I V E M K V L C K V H H
1276  GCCAGTCTGGTAGAGTTGATTGGCTATGCAGCAAGCAAGGATGAACTATACCTTATTTACGAGTACTCTCAAAAA
426   A S L V E L I G Y A A S K D E L Y L I Y E Y S Q K
1351  GGTTCACTCAAGAATCACCTTCATGATCCTCAAAGCAAAGGTTACACATCATTGTCTTGGATCTACAGGGTCCAA
451   G S L K N H L H D P Q S K G Y T S L S W I Y R V Q
1426  ATTGCTCTTGATGCGGCTAGAGGACTTGAATACATTCATGAGCATACAAAGGATCATTATGTTCACAGGGACATC
476   I A L D A A R G L E Y I H E H T K D H Y V H R D I
1501  AAATCGAGTAACATCTTGCTCGATGAATCCTTCAGAGCAAAGATTTCAGATTTTGGTCTTGCAAAACTGGTAGTG
501   K S S N I L L D E S F R A K I S D F G L A K L V V
1576  AAGTCAACTGATGCAGAGGCTTCTGTCACAAAAGTTGTGGGTACATTTGGTTACTTGGCCCCTGAATACTTGCGC
526   K S T D A E A S V T K V V G T F G Y L A P E Y L R
1651  GATGGCCTGGCAACTACGAAAAATGATGTTTATGCTTTTGGAGTAGTACTTTTTGAGTTAATATCTGGAAAGGAG
551   D G L A T T K N D V Y A F G V V L F E L I S G K E
1726  GCAATTACAAGGACTGATGGATTGAACGAAGGATCAAACTCAGAAAGGCGTTCCTTGGCATCAGTTATGTTGAGT
576   A I T R T D G L N E G S N S E R R S L A S V M L S
1801  GCACTAAAGAATTGTCGTAACTCAATGTATATGGGGAGCCTGAAAGACTGCATTGACCCTAATCTAATGGACCTG
601   A L K N C R N S M Y M G S L K D C I D P N L M D L
1876  TATCCACATGACTGTGTATACAAGATGGCCATGCTAGCGAAACAGTGCGTGGAGGAAGATCCTGTTCTTCGGCCA
626   Y P H D C V Y K M A M L A K Q C V E E D P V L R P
1951  GACATGAAGCAGGCAGTAATCACGCTATCGCAAATACTGCTGTCGTCGATCGAGTGGGAAGCGACGCTAGCTGGG
651   D M K Q A V I T L S Q I L L S S I E W E A T L A G
2026  AACAGCCAAGTGTTCAGTGGTCTTGTGGCTGGGAGGTGA
676   N S Q V F S G L V A G R *
```

FigureS1*OsRLK3* cDNA coding sequence and its deduced amino acid sequence

<u>ATG</u>: translation starting signal; <u>TGA</u>: translation termination signal; deduced amino acid sequence were marked in dark gray; red word: signal peptide; pink word: transmembrane domain; yellow word: LysM domain; green word: protein kinase domain.

```
1                                    AAGCAGTGGTATCAACGCAGAGTA
25      TTGGTGTCACAGAACCGTTGCAATGCGTTGACATTCGGTAACTTCTCTTCGACCAAATTC
85      AAACTCACCAAATGCGATGCAATCCGGAGAATGTAAGATTTTCGAGAGTCGGCTGCATCT
145     TTTGCCGAACTCATGACCTCCGATGAAAACTGAAATTCCGAACGTTAACAGTAGTGATGA
205     GAGGACCAATTAACAGATTTCAAACGGAATCCTGATGATAACTCGTTGTTCACGACCCTC
265     GAACGGCATGGCAACAAGCCGCAAAGAGGCCCCAACAACTCCTATAACTCTATGGCAACC
325     GTCGAACCAGCTAGCTGTTTGGTTTTCTTGCTGTTTCTTTCAGTAATTTCGAAATTAGCA
385     ATTTGATTCTACTTGAACTGTGAGATGACTTTCGAAACGTGAGAATTTTCAACCTTTTTT
445     GTTCTAAACTAAATTTTCAGTGAGAAGCTGAGAAGCGAGAAGAGAAACTCTCGGAGCGAT
505     CGTGCCTGTTCGGCACGTTGCTCTTCTCCGCGTCCGTATGGTCGATCGGCTTTCGTCCTT
565     TTCTCGTGGACAAGAATTTCGTTGAGTTAGACGTGATTTTGTTTTCTGATTTCCGACTTT
625     CTGTGACCAAAAATTTGCGAAACGTGATTCCACACCTTCTTTACGACCGTTTTCAGCGAA
685     ATGTCCGCAAGCCGTGCCGCCGGTGTTCCGCGCAAATACGCGACCAGTTTGTATCAGGCG
1        M  S  A  S  R  A  A  G  V  P  R  K  Y  A  T  S  L  Y  Q  A
745     GCAAAAAAACTGAACAAGTTGGACGCTGTGGAAAAGGACGTGAAGATCGTAAAAGATTTG
21       A  K  K  L  N  K  L  D  A  V  E  K  D  V  K  I  V  K  D  L
805     TACGCGTCTGATCAGAAGTTTTCGGCGTTTGTCAAGAATCCGACGTTGAATCGCAACTTG
41       Y  A  S  D  Q  K  F  S  A  F  V  K  N  P  T  L  N  R  N  L
865     AAACAGTCGGCGTTGACGAGTGTTCTGAAGTCAATTGGCGTTTCCTCGGATACGCAAAAA
61       K  Q  S  A  L  T  S  V  L  K  S  I  G  V  S  S  D  T  Q  K
925     TTCTTCGGAGTTTTGGCTGAAAATGGACGACTTGGATTTTTAAACGAGGTGCTCGTCAAC
81       F  F  G  V  L  A  E  N  G  R  L  G  F  L  N  E  V  L  V  N
985     TTCGAGGACATTCTCCGTTCCAACCGCGGCGATTTGACCGTCGAAGTTGTTTCGGCCGAC
101      F  E  D  I  L  R  S  N  R  G  D  L  T  V  E  V  V  S  A  D
1045    GCGTTGAACGACGCCACCAAACGCTCCATCAGCGATGCACTCGGAAAGAGTAGTAAGAGC
121      A  L  N  D  A  T  K  R  S  I  S  D  A  L  G  K  S  S  K  S
1105    GTCTCGATCACGTATCAGGTTCGACCGGAGATCATGGGCGGCTTGATCGTTCAGATTGGC
141      V  S  I  T  Y  Q  V  R  P  E  I  M  G  G  L  I  V  Q  I  G
1165    GATCGTCGTTTGGATCTGTCGATCGCATCGCGTGTCAAGAAACTCAACGAGACCATCAAA
161      D  R  R  L  D  L  S  I  A  S  R  V  K  K  L  N  E  T  I  K
1225    GAGGCCGTTTAATGATAGGGATGTTCGTCAGTAGGAACTCGTAGACTGTGTGATTATTTG
181      E  A  V  *  *  *
1285    GAGAAATAAAAATATAAACTTGTCGTGTGCCAAAAAAAAAAAAAAAAAAAAAAAAAA
```

Figure S2 *Ab-atps* cDNA sequence and its deduced amino acid sequence

ATG: translation starting signal; TAA: translation termination signal; deduced amino acid sequence were marked in dark gray.

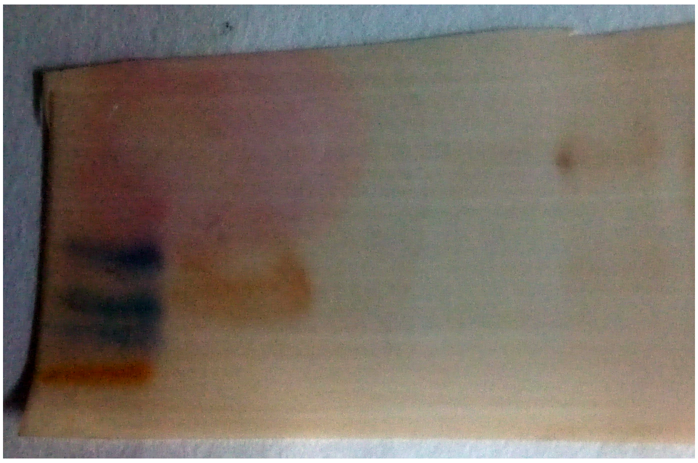

Figure S3 Figure of western blots used in Figure 9
